# Psychometric properties of the Chinese version of quality of life in life-threatening illness-family carer version

**DOI:** 10.3389/fpsyg.2022.925062

**Published:** 2022-08-03

**Authors:** Yitao Wei, Huimin Xiao, Hong Wu, Binbin Yong, Zhichao Weng, Weiling Chen

**Affiliations:** ^1^School of Nursing, Fujian Medical University, Fuzhou, China; ^2^Department of Hospice Care, Fujian Provincial Hospital, Fuzhou, China

**Keywords:** cancer, caregivers, quality of life, psychometrics, palliative care

## Abstract

**Background:**

The Quality of Life (QOL) in Life-threatening Illness-Family Carer Version (QOLLTI-F) has been proven to be a brief, reliable, and valid instrument for measuring the caregivers’ QOL in western cultures. However, whether it is suitable to be used in Chinese culture is unclear. This study aimed to test the reliability and validity of the Chinese version of (QOLLTI-F-CV).

**Materials and methods:**

A total of 202 family caregivers (FCs) of advanced cancer patients from Fujian Provincial hospice care center were investigated using the Chinese version of QOLLTI-F-CV from September 2019 to August 2020. The questionnaire was evaluated using an exploratory structural equation model. Its psychometric properties were examined in terms of factor structure, convergent validity, discriminant validity, internal consistency, and test–retest reliability.

**Results:**

Differently from the seven-domain original QOLLTI-F, its Chinese version had only three domains including caregiver’s self-feelings, caregiver’s stress, and caregiver’s outlooks. The total variance explanation rate for the domains was 55.4%. The Chinese version fitted well with the structure model (χ^2^ = 153.932, *df* = 75, *P* < 0.001); its comparative fit index (CFI) was 0.971; Tucker–Lewis index was 0.954; and the root mean square error of approximation (RMSEA) was 0.072. The success rate of its convergent and discriminant validity calibration test was 100%. Its Cronbach’s alpha coefficient of the whole questionnaire and three domains was from 0.650 to 0.874, and test–retest reliability was 0.836.

**Conclusion:**

The 3-domain QOLLTI-F-CV is a valid and reliable instrument for identifying QOL concerns of FCs of advanced cancer patients in China. The refactoring structure optimally matches Chinese culture and value system well.

## Introduction

The incidence and mortality of cancer are growing rapidly. The International Agency for Research on Cancer has reported that there were 19.3 million new cases of cancer and almost 10 million deaths from cancer in 2020. Among them, China accounted for 23.4% of the new cancer cases and 30.1% of the cancer deaths, which are ranked number one worldwide (Sung et al., 2021). Cancer puts burdens not only on cancer patients themselves but also on their family caregivers (FCs), especially in China, a family tie country. FCs assist patients in daily living activities, diet preparation, symptom management, and emotional support, which may interfere FCs’ own normal life and work (Geng et al., 2018; Teixeira et al., 2019). Meanwhile, FCs have to deal with their lovers’ imminent death. Oechsle et al. (2019) have revealed that FCs even experience more psychosocial burdens than cancer patients. Furthermore, their poor quality of life (QOL) could negatively impact the QOL of cancer patients (Sun et al., 2019; Lin Y. et al., 2020). Thus, it is necessary and important to assess the QOL of FCs.

Reliable and valid tools are essential to identify the QOL concerns of FCs of cancer patients. In China, some general QOL scales are available to measure FCs’ QOL, such as the *World Health Organization Quality of Life short version* and the *MOS item short from health survey* (Yu et al., 2017; Wang Y. et al., 2021). At oversea, some specific QOL scales have already been designed for FCs of cancer patients (Cohen et al., 2006; Lafaye et al., 2013). Among them, the *Quality of Life in Life-threatening Illness-Family Carer Version* (QOLLTI-F) is highly recommended. It was initially developed based on the seven themes that emerged from the qualitative research by Cohen et al. (2006), including environment, patient condition, carer’s own state, carer’s outlook, relationships, quality of care, and financial worries. It is unique that caregivers’ perception of patients’ conditions was included to attest to their close relationship. It not only covers the core attributes of the concept of QOL but also reflects the actual QOL of FCs. Additionally, it has only 16 items and seldomly increases the investigation burden of caregivers (Schur et al., 2014; Sawatzky et al., 2018).

The QOLLTI-F, originally designed in English and French, has been translated into several languages, such as German, Malaysian, Indian, Czech, Chinese, Swedish, Spanish, and Persian (Alnjadat et al., 2014; Nayak et al., 2014; Schur et al., 2014; Bužgová et al., 2015; Xiao et al., 2015; Axelsson et al., 2020; Arias-Rojas et al., 2021; Fereidouni et al., 2022). Previous studies have indicated that the QOLLTI-F may produce various domains under different cultural backgrounds. For example, Alnjadat et al. (2014) translated the QOLLTI-F into Malay and captured seven domains after forced extraction by exploratory factor analysis (EFA). However, only three of the seven domains totally complied with the original QOLLTI-F. Given the weak factor structure of the QOLLTI-F, Schur et al. (2014) performed a series of EFA, which cleanly supports a four-factor structure in the German version, in terms of feelings about carers’ own life, professional care, interaction with the patient and others, and carers’ outlook on life. But they pointed out that there was problematic cross-loading in some items in the factor analysis, and some farfetched explanations for the attribution of some items in the domains (Osborne and Costello, 2009; Schur et al., 2014). Similarly, Arias-Rojas et al. (2021) failed to replicate the original structure of the scale but obtained a new three-factor structure. They named the extracted three factors as impact of caregiving, social and health interactions, and measuring of life. Additionally, Fereidouni et al. (2022) also used the EFA method to extract three factors and employed confirmative factor analysis (CFA) to verify the structure. The three factors are caregiver’s physical emotional status, satisfaction with the situation, and caregiver’s concerns. But this population is the caregivers of patients with COVID-19, not the caregivers of cancer patients. To date, some other versions, such as the Swedish and Chinese versions, have not been validated so far, which may greatly hinder their application. In particular, considering that Chinese culture is quite different from western culture. This study, therefore, aimed to validate the Chinese version of (QOLLTI-F-CV) among FCs of advanced cancer patients in China.

## Materials and methods

### Study design and participants

A cross-sectional study was conducted in a hospice care center in Fujian Province, China. The sample size was calculated according to the ratio of participants to items at least 10:1 (Pett et al., 2003). A total of 214 FCs of advanced cancer patients were recruited for this study. The inclusion criteria were as follows: (1) age ≥18 years; (2) able to communicate with Mandarin Chinese; (3) the primary FC of cancer patients with less than 6-month life expectancy, who could be parents, adult children, spouses, or siblings; and (4) if there were several primary caregivers, the patient was responsible for identifying the primary one. The exclusion criteria were as follows: (1) not able to communicate and (2) cognition impairments (SPMSQ ≥3; the Short Portable Mental Status Questionnaire) (Pfeiffer, 1975).

### Instrument and measures

#### Personal information form

Personal information of the FCs was recorded, including gender, age, marital status, education, self-perceived health status, and relationship between patients and caregivers.

#### The quality of life in life-threatening illness-family carer version

The original QOLLTI-F was developed by Cohen et al. (2006) with 16 items and seven domains, including environment, patient condition, carer’s own state, carer’s outlook, relationships, quality of care, and financial worries. Its Cronbach’s alpha was 0.85, and test–retest reliability was 0.77. Pang et al. translated the QOLLTI-F-CV through the cross-cultural adaption and item analysis process (Xiao et al., 2015). The responses to every item were provided with a five-point score system ranging from 0 to 4 (0 = strongly disagree and 4 = strongly agree).

### Procedure

Study data were collected by two research assistants from September 2019 to August 2020. After obtaining the permission of Prof. Cohen, the author of the original QOLLTI-F, and Prof. Pang, the translator of the Chinese version, the physician from the study setting screened and referred the eligible participants to the research assistants. Then, the research assistants introduced the study and invited the eligible participants to fill in the questionnaires with informed consent. For participants with literacy difficulties, a research assistant read each item to them and then wrote down their oral responses objectively. At the beginning of the survey, the data were individually collected at the hospice care clinic face to face. Totally, 102 out of 105 valid questionnaires were gathered. Due to the COVID-19 epidemic, the remained data were collected online *via* “powered by www.wjx.cn.” In total, 100 out of 109 valid questionnaires were collected. The online survey quality was monitored by checking the time that FCs finished the questionnaires. The total of valid questionnaires was 202, with a response rate of 94.4% (202/214). According to Li’s study (Li, 2016), 30 FCs were invited to explore the test–retest reliability of the QOLLTI-F-CV after 2 weeks of the first survey.

### Analysis

Data input, processing, and statistical analysis were performed using IBM SPSS version 25.0. The exploratory structural equation modeling (ESEM) was conducted using Mplus version 7.0. The continuous missing values were replaced by the mean substitution (Streiner et al., 2015). The corrected item-total correlations and the Cronbach’s alpha if the item was deleted were computed for the item analysis. Corrected item-total correlations of 0.20–0.80 were considered satisfactory (Kline, 1986).

Before ESEM, Bartlett’s test of sphericity and Kaiser-Meyer-Olkin (KMO) measure of sampling adequacy was used to inspect the data (Hair et al., 1995). The number of extraction factors was determined by parallel analysis (PA) of the data. Retained the factors that the actual eigenvalues obtained by principal component analysis (PCA) are greater than the mean random data eigenvalues generated by Monte Carlo simulation (O’Conner, 2000).

The weighted least squares with mean and variance adjustment estimator were used for structural equation modeling analyses with categorical variables (Beauducel and Herzberg, 2006). The model’s goodness-of-fit was established using the following cutoff criteria: χ^2^/*df* < 5.0; the comparative fit index (CFI) >0.90; the Tucker-Lewis index (TLI) >0.90; and the root mean square error of approximation (RMSEA) <0.08 (Hu and Bentler, 1999; Hooper et al., 2008).

In addition, the convergent validity and discriminant validity of the Chinese version were evaluated by correlation analysis. Validity was assessed using the Spearman’s correlation coefficient. *P* < 0.05 was considered significant. If the correlation coefficient between the item and its domain is greater than or equal to 0.4, the convergent validity calibration experiment is considered successful. If the correlation coefficient between the item and its latitude is greater than that with other domains, the discriminant validity calibration experiment is considered successful. If the success rate of the calibration experiment is more than 80%, it means that convergence or discriminant validity is good (Li et al., 2002). In addition, the correlation of the domains of QOLLTI-F-CV with the self-perceived health status was measured using a correlation coefficient (Krabbe, 2016).

Internal consistency analyses were evaluated using the Cronbach’s alpha, with a value greater than 0.7 considered to be satisfactory (Devon et al., 2007). In terms of test–retest reliability, the value of the test–retest interclass correlation coefficient (ICC) that exceeded 0.60 was considered good (Kurtz, 2017).

## Results

### Participant characteristics

In total, 202 participants participated with a mean age of 48.36 ± 13.64 years, of which 55.0% were women and 90.3% were married. The majority of the FCs were adult children (41.1%) and spouses (37.1%) of patients. The participants’ characteristics are presented in Table 1.

**TABLE 1:** Distribution of participant characteristics (*N* = 202).

Characteristics	Mean (SD)/Frequency (%)
Age	48.36 ± 13.64
**Gender**	
Male	91 (45.0%)
Female	111 (55.0%)
**Marital status**	
Unmarried	13 (6.4)
Married	183 (90.6)
Divorced or widowed	6 (3.0)
**Education level**	
Uneducated	9 (4.5)
Primary school	44 (21.8)
Secondary school	54 (26.7)
High school and technical secondary school	48 (23.8)
Undergraduate or above	47 (23.3)
**Self-evaluation of health status**	
Excellent	85 (42.1)
Good	96 (47.5)
Fair	20 (9.9)
Poor	1 (0.5)
**Relationship to the patient**	
Parent	27 (13.4)
Child	83 (41.1)
Spouse	75 (37.1)
Sibling	6 (3.0)
Other	11 (5.4)

### Item analysis

As shown in Table 2, the correlation coefficients of item-total correlations ranged from 0.232 to 0.680, except for item 1 care place and item 10 spirituality comforting. As deleting any item did not significantly increase the reliability of the QOLLTI-F-CV and “care place” and “spirituality comforting” might have a potential impact on the QOL of FCs, we retained both two items for further factorial validity.

**TABLE 2:** Exploratory structural equation modeling solution with three factors and Cronbach’s alpha coefficients of the Chinese version of the quality of life in life-threatening illness-family carer version (QOLLTI-F-CV).

Factor (no. of items)	Factor loadings			Cronbach’s alpha	Item-total correlation	Cronbach’s alpha if the item was deleted
	F1	F2	F3			
F1: Carer’s Self-feelings (6)				0.874		
5. have time to take care of my physical and mental health	**0.885**	0.019	−0.070		0.600	0.806
2. I still have the private space I need	**0.819**	−0.035	−0.092		0.511	0.813
6. I can think clear	**0.622**	0.002	0.309		0.567	0.811
7. I feel in physical wellbeing	**0.757**	0.195	−0.003		0.667	0.803
8. I feel in physical wellbeing emotional wellbeing	**0.682**	0.293	0.016		0.680	0.800
9. Being able to take care of patients makes me feel good	**0.433**	0.333	0.193		0.652	0.803
F2: Carer’s Stress (5)				0.772		
14. I have stress to get along with patient	−0.031	**0.895**	−0.213		0.519	0.813
15. I have stress to get along with other relatives	0.030	**0.761**	−0.316		0.399	0.823
3. The patient’s condition distressed me	−0.208	**0.733**	0.016		0.378	0.821
4. I have difficulty in controlling the arrangement of my life	−0.198	**0.755**	−0.003		0.394	0.820
16. My financial situation is very tense	0.029	**0.608**	−0.164		0.394	0.820
F3: Carer’s Outlooks (5)				0.650		
12. I agree with the care decision-making for patients recently	0.009	−0.007	**0.811**		0.232	0.827
13. The quality of health care I and my patients get is excellent	0.047	0.027	**0.786**		0.284	0.826
10. My outlook on life, beliefs or religion give me strength and support	−0.061	−0.148	**0.691**		0.075	0.838
11. I think life is meaningful	0.078	0.134	**0.640**		0.335	0.823
1. It’s appropriate to take care of patients at home	0.263	−0.162	**0.288**		0.149	0.831
Total QOLLTI-F-CV (16)				0.827		

For exploratory structural equation modeling (ESEM) solution with five factors, all parameter estimates are standardized, and *a priori* target loadings designed to measure each factor are in bold.

### Construct validity

**FIGURE 1:**
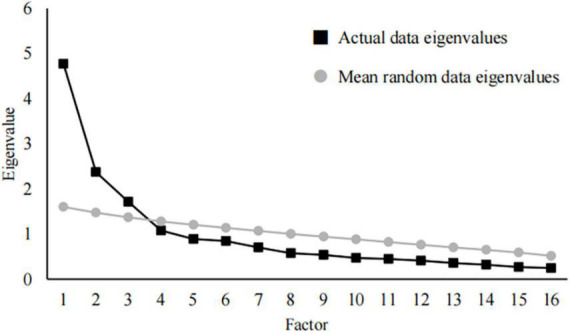
Actual data eigenvalues vs. mean random data eigenvalues.

The findings showed that Bartlett’s test of sphericity (χ^2^ = 1,191.160; *df* = 120) of the QOLLTI-F-CV was significant (*P* < 0.001), and the KMO was 0.821. Thus, all items were used for proceeding with PCA. Figure 1 presents the results from PA. The 16 items were grouped into three factors accounting for 55.371% of the total variance. The domains were entitled *carer’s self-feelings* (six items), *carer’s stress* (five items), and *carer’s outlooks* (five items). The approximate fit indices all indicated good model fit: χ^2^ = 153.932, *df* = 75, *P* < 0.001; CFI = 0.971; TLI = 0.954; and RMSEA = 0.072. However, item 1 (*It’s appropriate to take care of patients at home*) was problematic due to its factor load of <0.3. Considering its unique significance in FCs’ QOL, we kept it in the final questionnaire. The results from ESEM models are shown in Table 2.

### Convergent validity and discriminant validity

The correlation coefficient between the score of each item and the score of its domain was ≥0.4, which was higher than that between the score of this item and the score of other domains (*P* < 0.05). The achievement ratios of the convergent validity and the discriminant validity calibration test of the three domains were 100% (Table 3). Additionally, significant correlations were found among self-perceived health status and the “*carer’s self-feelings*” domain (*r* = 0.495, *P* < 0.001), “*carer’s stress*” domain (*r* = 0.192, *P* < 0.001), and total scores of QOLLTI-F-CV (*r* = 0.437, *P* < 0.001).

**TABLE 3:** The convergent validity and discriminant validity.

Domain	No. of items	Item convergent validity		Item discriminant validity	
		Range of correlations^a^	Rate of success^b^ (%)	Range of correlations^c^	Rate of success^d^ (%)
Carer’s self-feelings	6	0.681–0.852	100	0.089–0.412	100
Carer’s stress	5	0.400–0.854	100	0.001–0.400	100
Carer’s outlooks	5	0.463–0.747	100	0.016–0.266	100

^*a*^Correlations with own assumed domain. ^*b*^The correlation coefficient between items and assumed domain which is greater than 0.4. ^*c*^Correlations with other domains. ^*d*^The correlation coefficient between item and all domains which is significant.

### Reliability

The Cronbach’s alpha of the QOLLTI-F-CV was 0.827. The internal consistency of the three domains ranged from 0.650 to 0.874 (Table 2). The ICC of the test–retest measure was 0.836 for the total questionnaire.

## Discussion

This is the first study to examine the psychometric properties of the QOLLTI-F-CV. The findings support that it is a valid and reliable instrument for measuring the QOL of FCs of Chinese advanced cancer patients. Different from the original QOLLTI-F, our study suggests a three-factor structure solution for the Chinese population.

All 16 items of the original QOLLTI-F were retained in the QOLLTI-F-CV, but our study has revealed a stable three-factor solution. This is consistent with the results of the study by Arias-Rojas et al. (2021) and Fereidouni et al. (2022). Although some entries have a slightly different distribution of domains, we conducted PA to determine the number of factor extraction, which is more robust than the K1 method used by O’Conner (2000), Mu and Gu (2011), and Schur et al. (2014). Since ESEM has the advantage in terms of exploring factor structure flexibly and verifying the factor model systematically (Liu and Liu, 2013), it can make the model structure more consistent with the actual situation and the fitting more robust, compared with EFA which is used by Schur et al. (2014), Arias-Rojas et al. (2021), and Fereidouni et al. (2022). The results showed that the three-factor structured model fitted the empirical data well, as indicated by the fit indices. In addition, each item had good independence and representativeness.

Among the three domains of the QOLLTI-F-CV, domain 1 contains six items, which mainly reflects the FCs’ physical and mental endurance, and self-feelings toward care task (e.g., items 2, 5, 6, 7, 8, and 9), so it was named “carer’s self-feelings.” This domain contains items quite similar to the domain of “feelings about carers’ own life” in the German version and “caregiver’s physical emotional status” in the Persian version. When taking care of patients, FCs are endowed with a new role, which may compete and create conflicts with their other social roles (Yeung et al., 2020). Therefore, an in-depth understanding of FCs’ self-feelings is helpful for assessing their QOL. However, different from the German version, items 3 (*The patient’s condition distressed me*) and 4 (*I have difficulty in controlling the arrangement of my life*) are not included in this domain. It may be due to emotional suppression that is encouraged in Chinese culture (Chen et al., 2005). Furthermore, under the family norms of Chinese Confucianism, self-sacrifice is often made to provide “perfect” care for family patients. In the Persian version, items 2 (*I still have the private space I need*) and 9 (*Being able to take care of patients makes me feel good*) are classified as “satisfied with the situation.” Fereidouni et al. (2022) pointed out that this difference may be caused by the nature of the disease and the sample size.

Domain 2 entitled “carer’s stress” contains five items, which results from economic hardship, mental strain, and interpersonal relationship (e.g., items 3, 4, 14, 15, and 16). This is exactly the same as “caregiver’s concerns” in the Persian version. Compared with the original questionnaire, the German version, and the Spanish version, the Chinese version adds item 16 (*My financial situation is very tense*), item 14 (*I have stress to get along with patients*), and item 15 (*I have stress to get along with other relatives*) to “carer’s stress.” This could be explained by the following reasons. First, the financial situation is the most concern for Chinese families of advanced cancer patients (Xiao et al., 2015). In China, the cost of treatment and care for advanced cancer is regarded as a “bottomless pit” (Li, 2020; Zhou et al., 2020). Second, in many cases, FCs are not ready to take care of the dying patient. The closer relationship between FCs and patients, the more worried FCs are about patients, and the greater stress in facing the deterioration of their patients (Teng and Chen, 2013). It is reported that 96% of Chinese FCs have to reduce their working hours, and 72% even interrupt their work in order to take care of patients (Zhou et al., 2020). This not only has a great impact on their income but also leads to the limitation of their interpersonal activities (Shieh et al., 2012; Lin J. Q. et al., 2020). Generally, the stressors of Chinese FCs cover family finance and interpersonal relationship.

Domain 3 named “carer’s outlooks” contains five items, which reflect the FCs’ attitudes toward the role of care, medical decision-making, care quality, and personal value (e.g., items 1, 10, 11, 12, and 13). This domain not only includes the evaluation of caregivers’ care roles and personal values but also involves professional care. In China, medical staffs are the main consultants of patients and their families due to their professional authority (Soroka et al., 2021; Wang T. et al., 2021). In the process of caring patients, FCs frequently contact and communicate with medical staff, especially making a medical decision. Additionally, care quality also falls into the carer’s outlook domain. It may be related to Chinese strong family ties (Hou et al., 2018; Chung et al., 2021), which could explain why FCs much care about their patients. With regard to item 11 (*I Think Life is Meaningful*), except the Persian version, the German version, the Spanish version, and the Chinese version all suggest that it is not just a simple physical and emotional status, but more of a spiritual value. This may be related to the different effects of disease progression of cancer and COVID-19 on their caregiver’s awareness.

This study showed that the achievement ratios of the three domains of convergent validity and discriminant validity were all 100%. It showed that the items of each domain belong clearly and could distinguish each domain well (John and Benet-Martinez, 2000). Additionally, the better the self-perceived health status of caregivers, the better the QOL of FCs. The Cronbach’s alpha of the QOLLTI-F-CV was 0.827, and that of each domain was between 0.650 and 0.874. Only the Cronbach’s alpha of the “carer’s outlooks” domain was less than 0.7, but at least exceeded 0.6, which is considered satisfactory in practical research. The test–retest ICC of the scale was 0.836, indicating that it has good stability.

### Limitations

Several limitations in this study should be considered. First, the survey is carried out only in one hospice care center in Southeast China, which may affect the generation of the study results. The second limitation is that some respondents may be reluctant to express negative feelings related to care burden due to social expectation bias. Third, this study used on-site and online data collection due to the COVID-19 pandemic, which may affect the consistency and authenticity of data. Therefore, a multicenter with a larger sample survey could be conducted to copy the factor structure of the QOLLTI-F-CV in the future.

## Conclusion

This study provides evidence that the 3-domain QOLLTI-F-CV is a valid and reliable instrument. The refactoring structure optimally matches Chinese culture and value system well. It is a promising and accessible instrument for identifying QOL concerns of FCs of advanced cancer patients in China in clinical practices.

## Data availability statement

The raw data supporting the conclusions of this article will be made available by the authors, without undue reservation.

## Ethics statement

The studies involving human participants were reviewed and approved by the Human Ethics Committee of Fujian Medical University. The patients/participants provided their written informed consent to participate in this study.

## Author contributions

YW was responsible for the acquisition of data and manuscript drafting. HX was responsible for the conception and design of this study, critical revision of the manuscript, and supervision. HW was responsible for recruiting participants and supervision. BY was responsible for the analysis and interpretation of data. ZW and WC were responsible for recruiting participants and the acquisition of data. All authors read and approved the final manuscript.
